# Cutoff Point of HbA1c for Diagnosis of Diabetes Mellitus in Chinese Individuals

**DOI:** 10.1371/journal.pone.0166597

**Published:** 2016-11-18

**Authors:** Bing Wang, Ming-Chuan Liu, Xin-Yu Li, Xu-Han Liu, Qiu-Xia Feng, Lu Lu, Zhu Zhu, Ying-Shu Liu, Wei Zhao, Zheng-Nan Gao

**Affiliations:** 1 Department of Endocrinology, Dalian Municipal Central Hospital Affiliated Of Dalian Medical University, Dalian, China; 2 Graduate School, Dalian Medical University, Dalian, China; Florida International University Herbert Wertheim College of Medicine, UNITED STATES

## Abstract

**Background:**

The purpose of the present study was to find the optimal threshold of glycated hemoglobin (HbA1c) for diagnosis of diabetes mellitus in Chinese individuals.

**Methods:**

A total of 8 391 subjects (including 2 133 men and 6 258 women) aged 40–90 years with gradable retinal photographs were recruited. The relationship between HbA1c and diabetic retinopathy (DR) was examined. Receiver operating characteristic (ROC) curves were used to find the optimal threshold of HbA1c in screening DR and diagnosing diabetes.

**Results:**

HbA1c values in patients with DR were significantly higher than in those with no DR. The ROC curve for HbA1c had an area under the curve of 0.881 (95%CI 0.857–0.905; P = 0.000). HbA1c at a cutoff of 6.5% had a high sensitivity (80.6%) and specificity (86.9%) for detecting DR.

**Conclusions:**

HbA1c can be used to diagnose diabetes in a Chinese population, and the optimal HbA1c cutoff point for diagnosis is 6.5%.

## Introduction

Diabetes mellitus (DM), a disease that is defined by abnormal plasma glucose, has become widespread. The current diagnostic cutoff point in glucose levels for DM is based on the positive association between glucose levels and diabetic microvascular complications (particularly diabetic retinopathy) [1]. For decades, the diagnosis of DM in China was based on World Health Organization (WHO) criteria from 1999, including fasting plasma glucose (FPG) and 2-hour postprandial plasma glucose (2hPG) during a 75g oral glucose tolerance test (OGTT) [2].

Glycated hemoglobin (HbA1c) is a very important glycemic index that can be interpreted as indicating average blood glucose levels over the previous 3–4 months. HbA1c tests are convenient and can be performed at any time regardless of the time of the previous meal [3]. In Europe and the United States, an HbA1c ≥ 6.5% is used to diagnose diabetes [2, 4], but this cutoff is still controversial in China. Research evaluating HbA1c and its relevance to diabetes is very limited in China, and there is no unified standard for assessing HbA1c. HbA1c was not recommended for use in the diagnosis of diabetes in the China Guideline for Type 2 Diabetes published in 2013.

The objectives of this study were to reevaluate the relationship between HbA1c and diabetic retinopathy (DR), and to find the optimal HbA1c cutoff point for diabetes prediction in Chinese individuals.

## Materials and Methods

### Study population

This study was one part of the baseline survey from the REACTION study, which was a multicenter, prospective cohort study; its primary objective was to demonstrate whether abnormal glucose metabolism was associated with increased risk of cancer in a Chinese population [5, 6]. The study was approved by the ethics committee of Ruijin Hospital, Shanghai Jiaotong University School of Medicine. All participants provided written or verbal informed consent to participate in this study. From August to December of 2011, a total of 10 300 participants were recruited from two communities in the city of Dalian in Liaoning Province. Exclusion criteria were cancer, chronic liver disease, chronic kidney disease and glucocorticoid treatment; participants with incomplete data (especially digital fundus photographs and/or HbA1c analysis) were also excluded. In total, 8 391 subjects aged 40–90 years (2 133 men and 6 258 women) were eligible for the analysis. All participants were screened for DR by digital fundus photographs (CRNON CR6-45NM non-mydriatic ophthalmoscope digital camera, produced by Japan Cannon). Two photographs were taken of both eyes of each participant, according to the International Clinical Diabetic Retinopathy Severity Scales. Two qualified ophthalmologists from the department of ophthalmology in Dalian Municipal Central Hospital graded the photographs while blinded as to participants’ glucose and HbA1c levels. The degree of DR was determined according to the worse eye. When the two ophthalmologists were not in agreement, they consulted with an endocrinologist.

A standard questionnaire concerning health-related information and family history was used to obtain data for all participants by face-to-face interviews. All investigators received extensive training specifically for this questionnaire. All subjects had a physical examination including measurement of height, weight and waist circumference. Body mass index (BMI) was calculated as weight (kg) divided by the square of height in meters (m^2^). Waist-to-hip ratio (WHR) was calculated by dividing waist circumference (cm) by hip circumference (cm). Blood pressure was measured on the right arm three times consecutively at 1-min intervals, with the mean of the three measurements used for analysis. Blood samples were collected in the morning after at least 8 h of overnight fasting to determine glucose concentrations, HbA1c, serum uric acid (SUA) levels and lipid profile including total cholesterol (TC), triglycerides (TG), high-density lipoprotein cholesterol (HDL-C) and low-density lipoprotein cholesterol (LDL-C). Subjects without diabetes history underwent a 75g OGTT, and the subjects with previously diagnosed diabetes underwent a standardized steamed bread meal test. Venous blood was collected to measure 2hPG after the 75 g glucose or standardized steamed bread meal was taken. We measured plasma glucose concentrations by the hexokinase method, and SUA was measured by the uricase method using an automatic biochemistry analyzer (ADVLA 2004, Siemens). Serum for lipid profiles was stored below −20°C, and whole blood for HbA1c analysis was stored below 4°C, with samples delivered to the Chemical Laboratory of Shanghai Ruijin Hospital by a professional cold chain express company within 3 weeks. TC was measured by colorimetric enzyme assay and TG were measured by the phosphoglycerol oxidase method. HDL-C and LDL-C were measured by homogeneous assay methods, all using biochemical immunity conjunctedly machine (Architect Ci16200, Abbott). HbA1c was determined by ion-exchange high-performance liquid chromatography using an automatic glycated hemoglobin meter (Variant II, Bio-Rad).

### Definition

According to 1999 WHO diagnostic criteria, DM was defined as FPG ≥ 7.0 mmol/L and/or 2hPG ≥ 11.1 mmol/L, impaired glucose regulation (IGR) was defined as 6.1 mmol/L ≤ FPG < 7.0 mmol/L and/or 7.8 mmol/L ≤ 2hPG < 11.1 mmol/L, and normal glucose tolerance (NGT) was defined as FPG < 6.1 mmol/ L and 2hPG < 7.8 mmol/L.

The DR severity of each eye was graded according to the International Clinical Diabetic Retinopathy Severity Scales [7].

### Statistical Analysis

The Kolmogorov—Smirnov test was used to test whether continuous variables followed a normal distribution. Variables that were not normally distributed were log-transformed to approximate normal distribution for analysis. Quantitative variables were expressed as mean±standard deviation (SD), and qualitative variables were expressed as numbers and percentages. Comparisons between DR and no DR (NDR) groups were performed using two independent samples t-test, and for categorical variables, the chi-square test was used. Pearson correlation analysis test was used to analyze the relationship between HbA1c and FPG or 2hPG. Factors associated with DR prevalence were evaluated using multivariable logistic regression models. The receiver operating characteristic (ROC) curve was used to identify the best cutoff of HbA1c to balance the sensitivity and specificity for detecting DR. SPSS17.0 software (SPSS Inc., Chicago, IL, USA) was used for data analyses. A P value of <0.05 was considered to indicate statistical significance.

## Results

### Baseline characteristics

The final dataset included 8 391 participants (2 133 men and 6 258 women). According to the degree of DR in fundus photos, participants who had at least one eye with either non-proliferative diabetic retinopathy or proliferative diabetic retinopathy were assigned to the DR group for analysis; participants without DR were assigned to the NDR group (Table 1). HbA1c levels in the DR group were much higher than those in the NDR group (8.13±2.04% vs 5.98±0.92%; P = 0.000).

**Table 1:** Baseline characteristics for subjects.

Variables	Total	NDR group	DR group	P value
Number	8391	8138	253	
Hypentension (%)	16.3	15.6	40.5	0.000
Age(year)	57.29±8.02	57.14±7.95	61.86±8.63	0.000
BMI(kg/m^2^)	25.66±3.48	25.64±3.47	26.22±3.65	0.008
WHR	0.89±0.07	0.89±0.07	0.92±0.07	0.000
SBP(mmHg)	140.00±21.27	139.57±21.06	153.55±23.54	0.000
DBP(mmHg)	80.14±11.69	80.07±11.63	82.31±13.41	0.009
TC(mmol/L)	5.47±1.05	5.47±1.05	5.59±1.22	0.140
TG(mmol/L)	1.54±1.08	1.53±1.06	1.90±1.54	0.000
HDL-C(mmol/L)	1.41±0.32	1.41±0.32	1.30±0.30	0.000
LDL-C(mmol/L)	3.27±0.87	3.27±0.87	3.33±0.90	0.377
SUA(μmol/L)	307.85±86.40	307.65±86.65	314.35±77.80	0.18
FPG(mmol/L)	6.20±1.87	6.08±1.68	9.92±3.41	0.000
2hPG(mmol/L)	8.52±4.35	8.26±4.00	16.98±6.21	0.000
HbA1c (%)	6.04±1.04	5.98±0.92	8.13±2.04	0.000

### Prevalence of DR among different blood glucose group

According to their history of diabetes and the results of OGTT, subjects were divided into four groups: the NGT group (n = 4 527), the IGR group (n = 2 055), the newly diagnosed T2DM (NDM) group (n = 940) and the previous diabetes (PDM) group (n = 869). Group HbA1c levels were 5.65±0.34%, 5.86±0.38%, 6.75±1.36% and 7.79±1.73%, respectively (F = 2 416.78; P = 0.000). The prevalence of DR was 25.8% in the PDM group, 1.0% in the NDM group, 0.6% in the IGR group and 0.2% in the NGT group (χ^2^ = 1 719.802; P = 0.000; Table 2).

**Table 2:** Prevalence of DR (%).

Group	Total (cases)	NDR (cases)	DR (cases)	Prevalence of DR (%)
NGT	4527	4520	7	0.2
IGR	2055	2042	13	0.6
NDM	940	931	9	1.0
PDM	869	645	224	25.8

### Correlation between HbA1c and blood glucose levels

FPG and 2hPG were positively correlated with HbA1c (r = 0.83 and r = 0.795, respectively; both P = 0.000; Table 3).

**Table 3:** The analysis of the correlation between HbA1c and FPG or 2hPG.

Variable	r	P value
FPG	0.83	0.000
2hPG	0.795	0.000

### Odds ratios of DR risk factors

Factors associated with DR prevalence were evaluated using multivariable logistic regression models that included the following variables: age, diabetes duration, BMI, WHR, FPG, 2hPG, HbA1c, systolic pressure, diastolic pressure, HDL, LDL, TC, TG and SUA. Results showed that HbA1c (OR = 1.213, 95%CI 1.106–1.331), diabetes duration (OR = 1.055, 95%CI 1.028–1.082) and diastolic pressure (OR = 1.023, 95%CI 1.009–1.037) were significantly related to a higher risk of developing DR (Table 4).

**Table 4:** Independent factors associated with prevalence of DR.

	β value	odds ratio	95%CI	P value
HbA1c	0.193	1.213	1.106–1.331	0.000
diabetes duration	0.053	1.055	1.028–1.009	0.000
Diastolic pressure	0.023	1.023	1.009–1.037	0.001

### Utility of HbA1c for predicting DR

**Fig 1:**
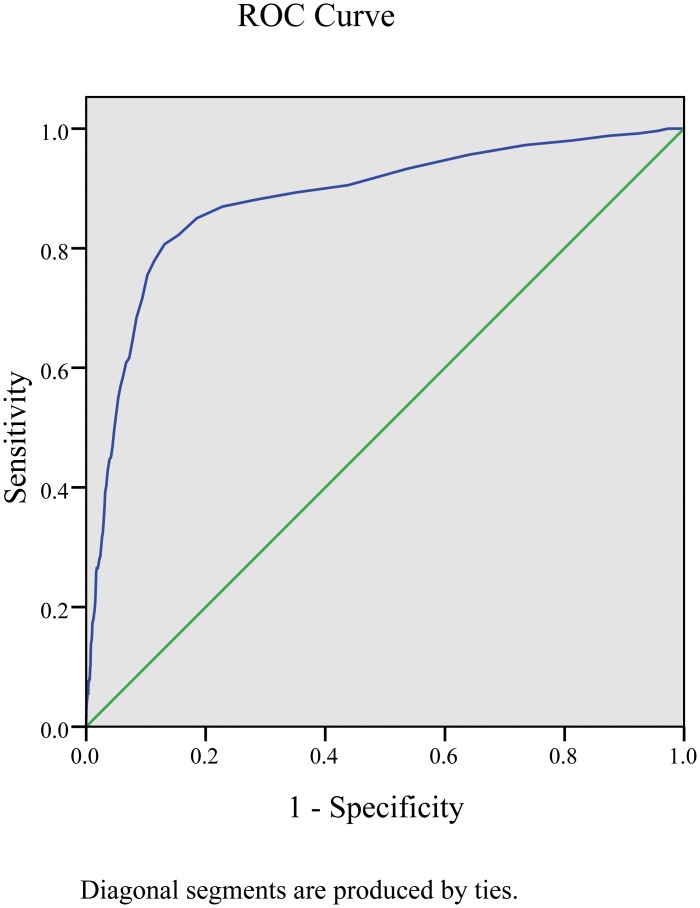
ROC curve analysis for the ability of HbA1c for detecting the presence of DR.

HbA1c was a highly predictive factor for detecting DR (S1 Dataset); the area under the ROC curve was 0.881 (95%CI 0.857–0.905; P = 0.000), and with the largest Youden index of 0.675, the optimal cutoff was 6.5%, with sensitivity of 80.6%, specificity of 86.9%, positive predictive value of 16.1% and negative predictive value of 99.3% (Fig 1 and Table 5).

**Table 5:** Sensitivity, Specificity, Positive predictive value, Negative predictive value and Youden index for detecting DR at different HbA1c thresholds by ROC curve.

HbA1c level (%)	Sensitivity (%)	Specificity (%)	Positive predictive value (%)	Negative predictive value (%)	Youden index
6.2	87.0	77.2	10.6	99.5	0.642
6.3	85.0	81.5	12.5	99.4	0.665
6.4	82.2	84.5	14.2	99.3	0.667
6.5	80.6	86.9	16.1	99.3	0.675
6.6	77.9	88.6	17.5	99.2	0.665
6.7	75.5	89.7	18.6	99.2	0.652
6.8	71.5	90.6	19.2	99.0	0.621

## Discussion

In line with extremely rapid economic growth and changes in lifestyle and dietary factors, T2DM is becoming a very serious public health problem worldwide, especially in developing countries [8]. The Chinese Diabetes Society reported that in China, one of the biggest developing countries, the prevalence of diabetes has increased dramatically over the past few decades, from approximately 1% in 1980 to 9.7% in 2010 [9].

Although the prevalence of diabetes has increased dramatically in recent years in China, many patients are diagnosed late [9, 10]. Diagnosis is often delayed until the appearance of complications; as up to 25% of people already have microvascular complications at diagnosis, many of them might miss the best timing of treatment, aggravating the financial burden on the country. Therefore, to identify diabetes in high-risk subjects in a timely manner is of utmost importance for the health care system.

Blood glucose levels have a continuous distribution, and as they become elevated, the risk for microvascular and macrovascular complications in diabetes increases sharply. Although the health and economic burden of T2DM is much more closely related to macrovascular than microvascular complications, there is no clear threshold for macrovascular complications. Currently, the criteria for diagnosing diabetes are based on the concept that as the risk of diabetes-associated microvascular complications (particularly DR) increases greatly above a threshold level of hyperglycemia, the threshold level can be used to differentiate people with diabetes [1, 11].

In China, T2DM is diagnosed according to 1999 WHO criteria. These criteria consider not only random or fasting glucose but also the results of OGTT. Although OGTT is regarded as the “gold standard” for the diagnosis of DM and IGR [12], it still has several limitations. First, OGTTs are time-consuming and unsuitable for large-scale screening. Second, there is poor reproducibility of the 2hPG during OGTT. Fasting is defined as no caloric intake for 8–10 hours; given the inconvenience of fasting and the special requirements for OGTT, another effective screening method to identify diabetes patients is needed.

HbA1c, a form of hemoglobin combining with blood glucose in red blood cells, can reflect the average plasma glucose concentration during the past three months. It is now widely used to monitor the long-term glycoregulation in treated diabetes [13]. Indeed, HbA1c has advantages over other indicators in the diagnosis and management of T2DM. First, HbA1c tests are convenient and can be performed at any time regardless of the time of previous meal. Second, HbA1c tests are highly reproducible and less invasive than OGTT [14]. In the current cross-sectional study, we demonstrated that FPG and 2hPG were positively correlated with HbA1c level. Epidemiological evidence has shown that the higher the HbA1c value, the higher the possibility of DR occurrence and evolution [15], and a similar relationship has been demonstrated for the FPG and 2hPG thresholds.

In 2009, the American Diabetes Association (ADA) and the European Association for the Study of Diabetes (EASD) all recommended using a threshold of HbA1c ≥ 6.5% to diagnose diabetes [4]; this recommendation was adopted by the ADA in 2010 [16]. Currently, the HbA1c test is widely used in Western countries [14]; however, there have been suggestions in China that HbA1c screening is inadequate because of lower sensitivity and non-standardized measurement nationwide [17]. Taken together with the fact that HbA1c may be affected by ethnicity and that there is a shortage of large scale epidemiological studies, this has meant that the clinical use of the HbA1c test in diagnosing diabetes in Eastern countries remains controversial. The China Guideline to T2DM in 2013 did not recommend HbA1c for diagnosing diabetes.

Recent studies indicated that compared with OGTT, HbA1c was similarly effective or superior in screening for or diagnosing diabetes. A meta-analysis was conducted to evaluate the diagnostic value of HbA1c ≥ 6.5% in Chinese diabetes patients and concluded that while the specificity of HbA1c ≥ 6.5% was relatively high, the sensitivity was low, and that other glucose tests were needed to reduce the false negative rate [18]. It is well known that ethnicity is related to differences in HbA1c thresholds and diagnostic efficiency [19, 20]. The Japan National Diabetes Survey reported an HbA1c threshold of 6.1% in detecting undiagnosed diabetes [2]. The fifth Korea National Health and Nutrition Examination Survey in 2011 recommended that the optimal threshold of HbA1c for predicting diabetes was 6.35% [21].

In China, results have been discordant in different areas and across different studies, and the optimal cutoff of HbA1c have varied dramatically. The Shanghai Diabetes Study II (SHDS II) in southern China found that an HbA1c cutoff of 6.3% could be accepted as a diagnostic criterion for diabetes [22]. A cross-sectional study in Karamay City in northwest China reported that an HbA1c threshold of 6.4% was the best cutoff point for diagnosis [23]. Another study in Qingdao City concluded that the optimal HbA1c threshold was 5.6% [24]. The Health Examination Survey in Beijing concluded that an HbA1c cutoff of 6.4% was highly specific and could be a good index for detecting undiagnosed diabetes in a Chinese population [25]. Inconsistencies in findings within individual studies led us to study this research question further in the current community-based study of over 8 000 middle-aged individuals. We examined the relationship between measures of glycemia and DR, and found that HbA1c levels were closely related to FPG and 2hPG levels. Further, HbA1c levels in the DR group were much higher than those in NDR group, and higher levels of HbA1c were associated with a higher prevalence of DR.

ROC curves are widely used in describing and comparing the performance of diagnostic technology. In this study, the area under the ROC curve was 0.881. The optimal cutoff value with the highest discriminant ability was 6.5%.

Compared with HbA1c ≥6.4% and 6.3%, the point of 6.5% had higher sensitivity (80.6%) and specificity (86.9%) for detecting DR and was consistent with the international standards. To further evaluate the specific value of HbA1c ≥ 6.5% in DR diagnosis, we divided participants into two groups: an HbA1c < 6.5% group and an HbA1c ≥ 6.5% group. The prevalence of DR was 0.7% and 16.1% respectively in these two groups. These data indicate that HbA1c ≥ 6.5% is a good cut point and if the HbA1c level exceeds this point, the prevalence of DR will increase dramatically.

Not surprisingly, this cutoff value (HbA1c ≥ 6.5%) is inconsistent with values from other studies done in China, such as ≥6.4% in the Beijing and Karamay studies, ≥6.3% in the Shanghai study and ≥5.6% in the Qingdao study; different demographic and biochemical characteristics of these studies are likely to have led to different results. First, while the participants in the Karamay study were all aged ≥40 years and had no history of diabetes, the subjects in Beijing were between 18 and 79 years old. Subjects in the Shanghai study were over 20 years of age, and subjects in the Qingdao study 35 years and older; our study population was middle-aged, with subjects 40 years and older. Second, the diabetes diagnostic criteria were different between studies; although the present study and the Beijing study used the presence of DR as an indicator of the presence of diabetes, the others all used OGTT as the diagnostic criterion.

Our study had some limitations. First, it was based on cross-sectional data. Second, it may be that non-glucose factors such as hemolytic anemia, iron deficiency anemia and hemoglobinopathy could influence the veracity of HbA1c detection. We could not exclude the influence of these conditions because hemoglobin concentrations and serum iron levels were not available.

Despite the controversies concerning its practical application, HbA1c is thought to be a useful tool in diagnosing diabetes. Our study examined the relationship between HbA1c and DR and found that HbA1c with the optimal cutoff of 6.5% might be effective in diagnosing diabetes in the Chinese population. This threshold is similar to that recommended by the ADA.

## Supporting Information

S1 Dataset (RAR)
